# Comparison of different spectral cameras for image-guided organ transplantation

**DOI:** 10.1117/1.JBO.26.7.076007

**Published:** 2021-07-24

**Authors:** Richard Mühle, Wenke Markgraf, Anna Hilsmann, Hagen Malberg, Peter Eisert, Eric L. Wisotzky

**Affiliations:** a Technische Universität Dresden, Institute of Biomedical Engineering, Dresden, Germany; b Technische Universität Dresden, Department of Neurosurgery, Faculty of Medicine Carl Gustav Carus, Dresden, Germany; c Fraunhofer Heinrich-Hertz-Institute, Department of Vision and Imaging Technologies, Berlin, Germany; d Humboldt Universität zu Berlin, Department of Visual Computing, Berlin, Germany

**Keywords:** hyperspectral imaging, multispectral imaging, image-guided surgery, organ analysis, blood analysis, spectral camera comparison

## Abstract

**Significance:** Hyperspectral and multispectral imaging (HMSI) in medical applications provides information about the physiology, morphology, and composition of tissues and organs. The use of these technologies enables the evaluation of biological objects and can potentially be applied as an objective assessment tool for medical professionals.

**Aim:** Our study investigates HMSI systems for their usability in medical applications.

**Approach:** Four HMSI systems (one hyperspectral pushbroom camera and three multispectral snapshot cameras) were examined and a spectrometer was used as a reference system, which was initially validated with a standardized color chart. The spectral accuracy of the cameras reproducing chemical properties of different biological objects (porcine blood, physiological porcine tissue, and pathological porcine tissue) was analyzed using the Pearson correlation coefficient.

**Results:** All the HMSI cameras examined were able to provide the characteristic spectral properties of blood and tissues. A pushbroom camera and two snapshot systems achieve Pearson coefficients of at least 0.97 compared to the ground truth, indicating a very high positive correlation. Only one snapshot camera performs moderately to high positive correlation (0.59 to 0.85).

**Conclusion:** The knowledge of the suitability of HMSI cameras for accurate measurement of chemical properties of biological objects offers a good opportunity for the selection of the optimal imaging tool for specific medical applications, such as organ transplantation.

## 1 Introduction

One of the largest problems for the transplantation medicine is the lack of donor grafts.^1^ Compared to 2010, the rate of postmortem organ donations in Germany has decreased by 29% in 2019, which corresponds to 1210 organs. Despite an improvement with respect to 2017, the need for organs in 2019 (9271 organs required) far exceeds the number of available donor organs (3767 organs transplanted).^2^ This high demand for donor organs shows in particular the need to increase the number of successfully transplanted organs. Optimal use of the few donor organs is urgently required not only with regard to the individual person to be treated but also from a social and political point of view. The most frequently transplanted organs are kidney, liver, heart, and lung. However, in Germany, the discrepancy between supply and demand in kidney transplantation is alarming. A total of 2291 kidneys has been available for transplantation in 2018, compared to 7526 open transplant recipients at the end of the year.^3^ Apart from this shortage, the rejection rate for available kidney transplants has been 14.8% in 2019.^2^ Due to the lack of an accurate predictive organ evaluation strategy, certain organs were rejected although they would still have been suitable for transplantation.^4^^–^^7^ The decision to accept or reject an organ for transplantation depends primarily on the expertise of the transplant team rather than on an objective measurement. In addition in the years 2014 to 2019, intra- or postoperative complications of kidney transplantations requiring a second kidney transplantation have been between 15.3% and 20.4%.^2^^,^^3^^,^^8^ Therefore, an optical evaluation tool to monitor organ quality before transplantation would be of great interest.

The digitization of intraoperative imaging procedures offers the possibility of both the analysis of acquired image data directly during the medical application and the visualization of the automatically extracted information for the surgeon. In this way, information about circulatory disorders, tissue types, or potential structures at risk are obtained during surgery. Hyper- and multispectral imaging (HMSI) are intended to expand the existing intraoperative imaging procedures with the aim of differentiating tissue types as precisely as possible.^9^^,^^10^ Spectral cameras record the reflected light components, which can be used to draw conclusions about the physiologically relevant parameters of tissue. Thus HMSI has the potential to measure the optical tissue behavior temporally and spatially resolved. This imaging technique operates among other spectral ranges in the region of the optical window (λ=600 to 1300 nm), where the main absorbers are melanin, lipids, water, and hemoglobin (Hb).^9^ For different biomedical areas, e.g., histology, wound healing, retinal diseases, or cancer detection, the potential of HMSI has been shown.^9^^,^^11^^–^^14^ It shows the feasibility of detecting variations in Hb at different oxygen saturation levels^14^^–^^17^ of mapping water content in organs^18^ as well as to evaluate organ blood flow characteristics.^19^^,^^20^

In organ preservation prior to transplantation, HMSI could be a potential tool to continuously monitor several functional parameters of the organ simultaneously and non-invasively.^16^^,^^19^^,^^21^^–^^24^ Such evaluation of organ quality could help to ensure the appropriate use of donor organs and increase the number of successful transplantations. To evaluate organ quality, different relevant spectral bands have to be analyzed, e.g., water band at λ=970  nm, oxygen band at λ=760  nm, and characteristic Q bands of present porphyrin complexes.^25^ Thus specific markers can be defined to specify organ quality. For example, tissue oxygen saturation (${\mathrm{StO}}_{2}$) is a promising marker of organ quality.^26^ Further, the optimal blood supply of a transplanted organ is essential for the therapeutic outcome. Currently, the monitoring of such functional parameters and ensuring homogeneous perfusion of a transplanted organ is usually associated with an increased diagnostic effort. This includes contact with the organ and a time-consuming measurement that provides only selective spatial and temporal information.^27^^,^^28^ A continuous and contactless monitoring of such parameter would make a significant contribution to organ assessment prior and during the transplantation.

For application in different medical fields, especially organ transplantation, HMSI systems have to operate robustly and in a wide spectral range. Therefore, this study compares and evaluates different types of spectral cameras for possible clinical use, such as organ evaluation during transplantation, examining four different porcine organs, and autologous whole blood of three pigs. Thus it can be shown that spectra can be reconstructed using these cameras, which produce useful results for biomedical applications. In the next section, the setup with all cameras as well as its calibration and the scanning process are described. Further, the analyzed organs with its removal and preparation process are presented. The optical properties and the different camera behaviors are presented in Sec. 3. The results of the study are discussed in Sec. 4.

## 2 Materials and Methods

### 2.1 Spectral Camera Specifications

Four spectral cameras have been used in this study, which are based on two different acquisition techniques. The specifications of each camera and its sensor are presented in Table 1. One camera is a hyperspectral pushbroom camera (Tivita Tissue 8, Diaspective Vision GmbH, Germany), which holds a CMOS sensor and a dispersive element. Each captured two-dimensional image consists of a spatial axis y and the wavelength information λ. During a scan process, 640 y-λ images are acquired along the spatial x axis, giving the third dimension of a complete data cube. The system is described in more detail by Mühle et al.^14^

**Table 1:** The sensor and camera optics specifications of the different spectral camera systems used. The last three parameters have been set during tissue analysis of the porcine organs. The specific bandwidths and spectral resolutions of the three snapshot mosaic cameras can be found in Table S1 in the Supplementary Material.

Camera	Pushbroom	Snapshot 4×4	Snapshot 5×5	Snapshot 3×3
Sensor	CMOS	CMOS	CMOS	CMOS
Sensor ADC (bits)	12	10	10	10
Sensor resolution (px)	1280×960	2048×1088	2048×1088	1280×1024
Output resolution (px)	640×480	2048×1024	2045×1080	1280×1024
Sensor size (mm)	4.8×3.6	11.3×6.0	11.3×6.0	6.8×5.5
Pixel size (μm)	3.75	5.5	5.5	5.3
Focal length	12 mm f/1.4	75 mm f/2.8	75 mm f/2.8	35 mm f/1.65
Spectral range (nm)	500 to 995	463 to 638	693 to 966	430 to 700
No. of bands	100	16(4×4)	25(5×5)	8+1(3×3)
Bandwidth (FWHM avg.) (nm)	5	15	15	40
Spectral resolution (nm)	5	8 to 12	8 to 12	27 to 41
Frame rate (fps)	60	20	20	12
Exposure time (ms)	0.25	50.0	50.0	83.3
WD (mm)	403	319	319	272

Three cameras are snapshot mosaic cameras using two different sensor setups. Two cameras (MQ022HG-IM-SM4X4-VIS and MQ022HG-IM-SM5X5-NIR, XIMEA GmbH, Germany) hold a 4×4- and a 5×5-mosaic pattern, which includes 16 and 25 wavelength bands, respectively. The 4×4-mosaic sensor is sensitive in the visible (VIS) spectrum while the 5×5-mosaic sensor is sensitive in the near-infrared (NIR) spectrum. The 5×5-mosaic camera contains a longpass filter in front of the optics to cut off all wavelengths λ<675  nm to avoid secondary bands. Thus each pixel in the mosaic pattern corresponds to one wavelength band. These two cameras are described in more detail by Wisotzky et al.^10^^,^^29^ The third snapshot camera (CMS-C, SILIOS, France) holds a 3×3-mosaic pattern with a slightly different setup. The center pixel of the mosaic pattern is sensitive in the complete sensor sensitivity range and behaves as intensity pixel, whereas the eight surrounding pixels (px) select specific wavelengths λ in the VIS spectrum. All three snapshot cameras can handle 60 fps or higher.

In addition to the four camera systems, a spectrometer has been used as a reference system for the analysis of tissue and blood samples. The spectrometer (USB2000 + VIS-NIR-ES, Ocean Optics Inc., USA) with its beam path (QR400-7-VIS-BX, Ocean Optics Inc., USA) is an asymmetrical Czerny–Turner monochromator using a CCD detector. It has a spectral resolution of 1/3  nm and covers a wavelength range of 350 to 1000 nm.

### 2.2 Imaging Setup

**Fig. 1:**
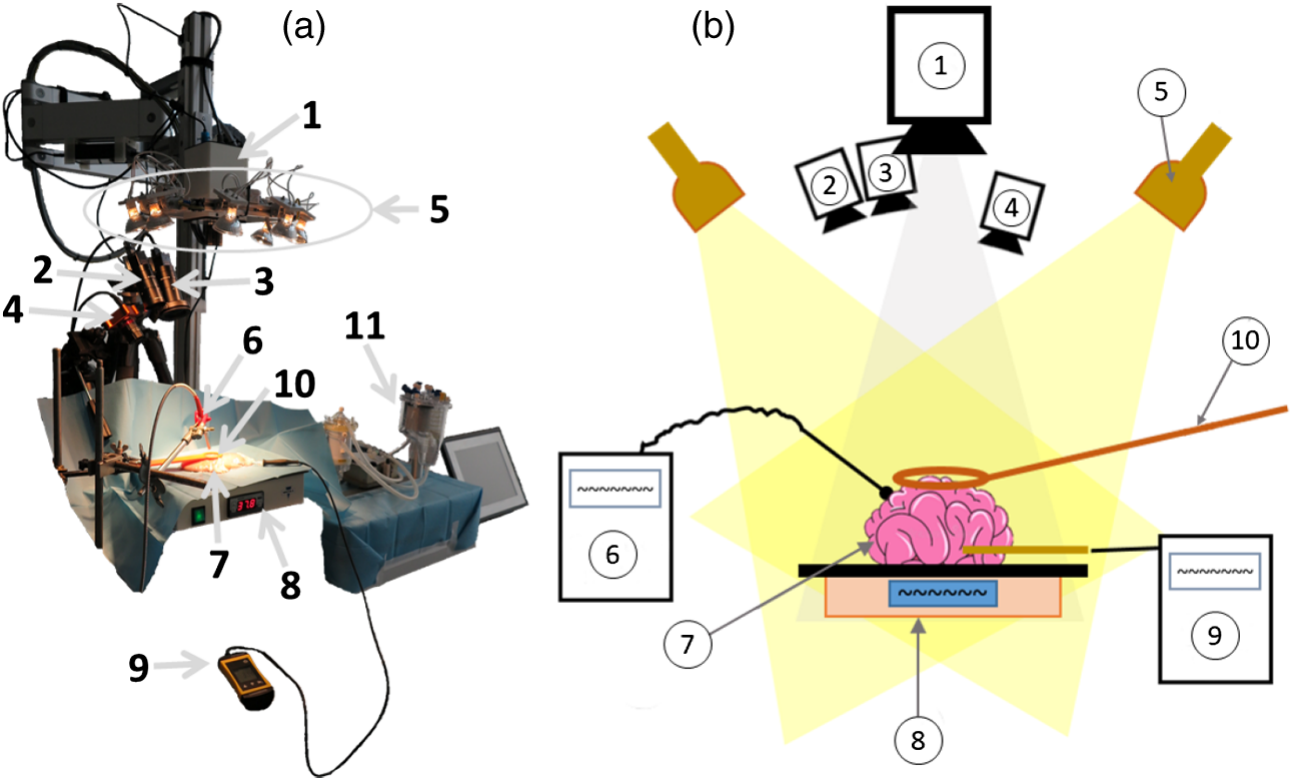
The setup is shown during (a) calibration and (b) as sketch with the complete equipment: (1) pushbroom camera setup, 41-bands setup with (2) 4×4-VIS camera and (3) 5×5-NIR camera, (4) 3×3-VIS camera setup, (5) six illumination spots, (6) spectrometer, (7) tissue sample, (8) heating unit, (9) thermometer, (10) cursor, and (11) perfusion machine for blood measurements.

The acquisition setup with all four cameras and illumination spots is shown in Fig. 1. The illumination unit contains six 20-W quartz-tungsten-halogen spots (OSRAM Decostar 51 ALU, Osram GmbH, Germany) with an aluminum reflector and a defined beam angle and beam position (Fig. 1, No. 5). Each spot has a luminosity of 510 cd and a color temperature of 2800 K. All spots are mounted in a distance of 450 mm to the target and combined having an illuminance of 12.2 klux in that working distance (WD).

All cameras (Fig. 1, Nos. 1 to 4) focus on the same target area, which is indicated by a cursor defining a circular region of interest (ROI) of d=30  mm (Fig. 1, No. 10). Snapshot cameras acquire the entire wavelength information in one shot resulting in a spatial/spectral 2D representation of the dataset but with a reduced spatial resolution and fewer spectral bands (8 to 25 for the used cameras in this study). As the two Ximea snapshot cameras (Fig. 1, Nos. 2 and 3) are mounted on one static plate (stereo-like) and cover different spectral intervals, i.e., λ=463 to 638 nm for the 4×4-VIS and λ=693 to 966 nm for the 5×5-NIR camera, respectively, the data of both cameras are combined to one spectral dataset using pixel correspondences between the two cameras calculating a disparity map^30^^–^^33^ (hereinafter referred to as multispectral 41-bands setup) and are then analyzed. The Silios snapshot camera (Fig. 1, No. 4) covers the same spectral interval as the 4×4-VIS camera and furthermore covers the gap between the two Ximea cameras. Hence, it is used as a comparable 3×3-VIS setup. This allows an estimation about the minimum required number of spectral bands (16 bands of 4×4-VIS versus 8 bands of 3×3-VIS) for robust organ surveillance. The pushbroom camera (Fig. 1, No. 1) captures the complete spectral dataset of a recorded slit in one shot. The second spatial component is created by moving the slit. The recorded data cube consists of 640 pixels in the traverse direction of the slit and 480 pixels in the direction of the slit. The angle between the illumination units and the different cameras is in the range of α≈25  deg to 35 deg. For reference data, a spectrometer (USB2000 + VIS-NIR-ES, Ocean Insight, USA) measures the object in the circular area (Fig. 1, No. 6). The spectrometer points toward a small region at the border of the circular area. As the spectrometer has no local resolution, the reference spectrum is averaged over the entire measurement area.

To obtain the reflectance spectrum from the measured raw data, each image has to be corrected according to

$$I_{\text{reflectance}}=\frac{I_{\text{raw}}-I_{\text{dark}}}{I_{\text{white}}-I_{\text{dark}}},$$ (1)

where $I_{\text{reflectance}}$ is the resulting reflectance spectrum, $I_{\text{raw}}$ is the measured raw data, $I_{\text{dark}}$ contains the dark reference data, and $I_{\text{white}}$ is the white reference intensity spectrum, as described in particular by Mühle et al.^14^ and Wisotzky et al.^10^^,^^29^

### 2.3 Organ and Blood Analysis

We have analyzed tissues of typical donor organs (heart, lung, and kidney) to compare the four introduced spectral cameras. In addition, brain and blood have been analyzed as well. All organs used in this study have been considered healthy by the veterinarian present at the time of collection. In addition, one kidney affected with cysts is collected. All organs are retrieved from three female German Landrace pigs weighting 100 to 120 kg. The cyst-affected kidney is retrieved from the first pig, which is the only pig where both kidneys, a healthy and a cyst-affected kidney, have been collected. After death by exsanguination, all organs and the blood have been collected. Approximately 1.5 l of blood has been received from the carotid incision into a receptacle containing 10,000 units of heparin (Rotexmedica GmbH, Trittau, Germany). The blood has been stored at 4°C. All other organs have been removed en bloc and, with exception of the brain, flushed immediately with preservation solution (Custodiol, Dr. Franz Köhler Chemie GmbH, Bensheim, Germany). Afterward, the organs have been placed into a bag with preservation solution and stored on ice at 4°C. For blood collection, the time between exsanguination and storing on ice has been ∼2  min. The process of organs and blood removal is described in more detail by Markgraf et al.^23^ The warm ischemia time occuring between interruption of blood supply and storage on ice has been ∼44.5±0.5  min for each porcine model. Before starting the measurement procedure, the organs have been removed from ice after ∼1 up to 3.5 h cold ischemia time (CIT) and are heated up to the target temperature of ∼37°C. The heating process has been carried out for 25 min in a water bath. The procedure described in this paragraph has been adapted to simulate a state-of-the-art clinical transplantation procedure, where the spectral measurement would be performed 25 min after organ reperfusion.

### 2.4 Measurement Procedure

At the beginning, reference measurements with a color chart (ColorChecker Classic, X-Rite Inc.) have been performed to set up the optimal camera and spectrometer settings, such as exposure time, focus, or frame rate, for each device (cf., Table 1). In addition, these measurements have been used to validate the spectrometer measurements as the colored tiles hold well-defined color spectra.^34^ For each colored tile, the reflectance spectrum is known in the range of 390 to 1000 nm with step size of 10 nm.^35^ Because of this validation, we can use the spectrometer measurements as ground truth or reference data for the organ measurements. The spectrometer measurements are performed by averaging 10 scans, a Boxcar width of 2, an integration time of 8 ms per scan, and a distance of 3.5 cm with angle of 12.5 deg to the target area having diameter of 1.95 cm. The software OceanView 1.6.3 (Lite, Ocean Optics Inc., USA) has been used to record and process the raw data.

Then all organs of each porcine model are measured successively in the order brain, kidney, lung, and heart. After each heating process, the organs are placed under the cursor (Fig. 1, No. 10) on a heating unit (Fig. 1, No. 8) to ensure a constant temperature during the measuring process. A thermal sensor (Fig. 1, No. 9) is placed inside the organs to control the core temperature during the measurements. For calibration purposes [cf., Eq. (1)], white and dark images are acquired before as well as after each organ measurement to achieve $I_{\text{white}}$ and $I_{\text{dark}}$, respectively. For acquisition of $I_{\text{white}}$, a white standard (99% reflectance Zenith, SphereOptics GmbH, Germany) is recorded simultaneously with all cameras and the spectrometer using the same illumination. Dark images are acquired after occluding the optics of all cameras and the spectrometer.

**Fig. 4:**
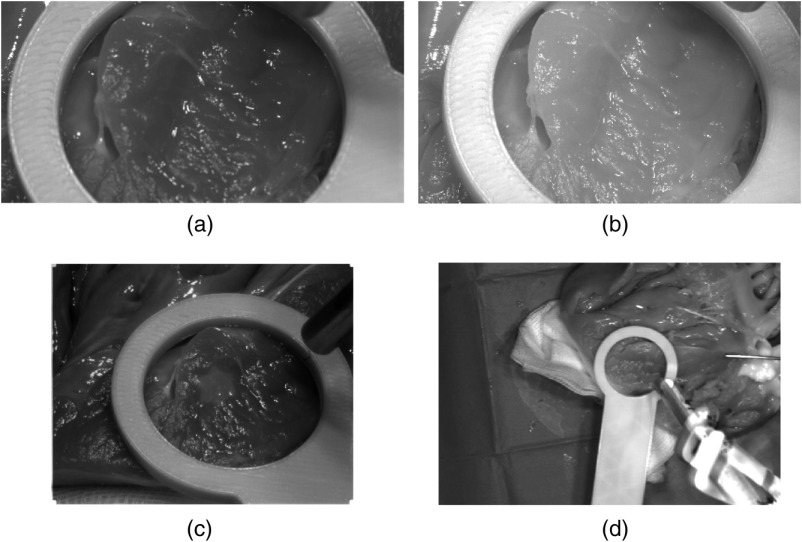
Spatially resolved HMSI images of the heart of the third pig model. (a) Multispectral 4×4 VIS camera; (b) multispectral 5×5 NIR camera; and (c) Multispectral 3×3 VIS camera. One spectral band of the snapshot sensor data for (a) λ=530  nm, (b) λ=746  nm, and (c) λ=515  nm. (d) The intensity distribution of the TIVITA Tissue pushbroom camera image at λ=750  nm.

For each organ as well as the blood samples, three measurements have been performed with all cameras simultaneously. Each camera captures the ROI bordered by the cursor (Fig. 1, No. 10; cf., Fig. 4). One pushbroom measurement takes about 10 s. In parallel, 10 images have been acquired for each of the snapshot cameras. These 10 images are averaged to one dataset. Four different healthy organs of three pigs, one additional kidney affected with cysts as well as blood with three different oxygenation conditions have been scanned. Consequently, a total of 48 measurements has been performed for each camera: 36 healthy organ measurements, 3 cyst-affected kidney measurements, and 9 blood measurements. The blood conditioning experiments have been carried out in accordance to Markgraf et al.^23^ and three different oxygenation saturation levels (98.6%, 92.8%, and 82.0%) have been set up. Since specular reflections are unavoidable in the ROI marked by the cursor, for the analysis, two smaller areas in the ROI are selected manually by biomedical experts considering the anatomical structures in each measured image of each camera, which contain no interfering artifacts. These two areas represent the similar location over all cameras per measurement. Each area has a minimum size of 21  px×21  px×N bands, where N is the number of possible wavelength bands dependent on the used camera. The measured sensor data of these ROIs are averaged for further evaluation in MATLAB R2019b (The MathWorks Inc., Natick, Massachusetts, USA).

## 3 Results

### 3.1 Color Checker Measurements

**Fig. 2:**
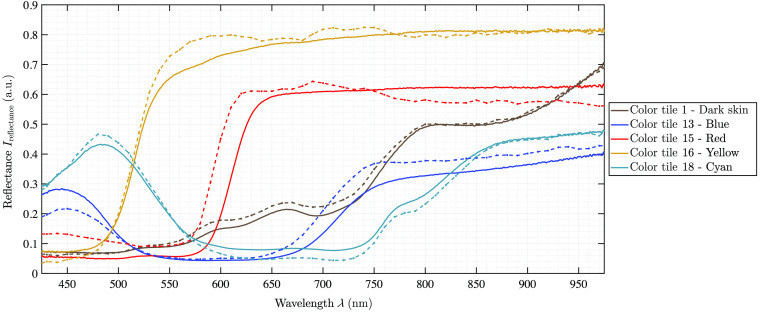
Five different analyzed color tiles of the Color Checker Board. The continuous lines are measured with a spectometer in this study and the dashed lines are published reference measurements.^34^^,^^35^

**Fig. 3:**
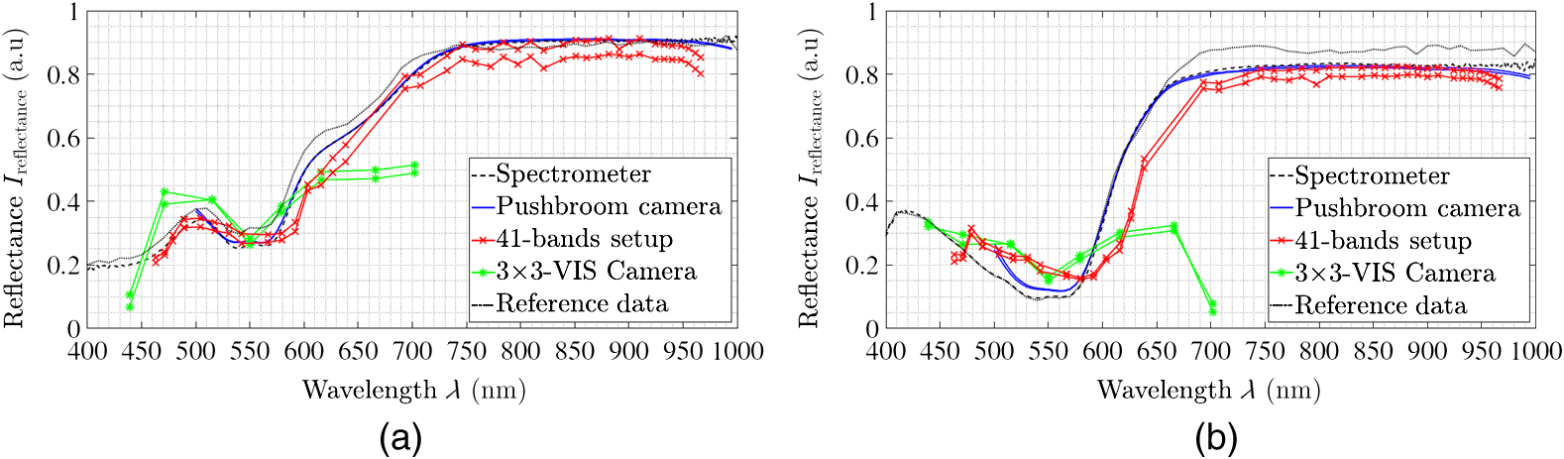
Two color tiles, with defined colors (a) light skin and (b) magenta, analyzed with all four described setups. The two curves per imaging setup correspond to the two selected ROIs in each captured image.

Reference measurements with the color chart are applied to validate the spectrometer measurements using published reflectance data of the colored tiles. Several characteristic color tiles, showing different spectral distributions, have been scanned. Five representative reflectance data are shown in Fig. 2. The comparison between the spectra of the spectrometer and the known spectra of the color chart shows comparable results. The highest deviation between these spectra is 5%. In addition, two further color tiles are measured with the three camera setups to evaluate the comparability of the achieved reflectance data on a standardized, non-biological test object. The data recorded simultaneously by the four spectral cameras as well as the spectrometer have slightly different formats. Four spectral curves are achieved from the measurements (cf., Table 1): (1) spectrometer reference data (λ=400 to 1000 nm with step size 1/3  nm), (2) hyperspectral pushbroom camera (λ=500 to 995 nm giving 100 bands in step size 5 nm), (3) multispectral 41-bands setup (λ=463 to 966 nm giving 41 bands), and (4) multispectral 3×3-VIS snapshot camera (λ=430 to 700 nm giving eight bands). The camera setup results of the two color tiles are shown in Fig. 3. The pushbroom camera data as well as the 41-bands setup data follow the published color spectra as well as the ground truth spectrometer data. The multispectral 3×3 camera data show more variability in the eight reconstructed data bands. In the following, these behaviors are presented and discussed in detail based on the organ measurements of the three analyzed pigs.

### 3.2 Organ Measurements

The measurements of the different porcine organs have been performed in a time range of approx. t=2  h, i.e., t=2  h to t=4  h after collection. Thus the CIT has been in the range of $t_{\mathrm{CIT}}=63\;\min$ and $t_{\mathrm{CIT}}=214\;\min$. The core temperatures $T_{\text{core}}$ of the healthy organs during the measurements are presented in Table 2. Organ core temperatures $T_{\text{core}}$ have ranged between $T_{\text{core}}=31.3{}^{\circ}C$ and $T_{\text{core}}=36.1{}^{\circ}C$.

**Table 2:** Organ core temperature $T_{\text{core}}$ (°C) recorded during the measurements of kidney, lung, heart, and brain in the three porcine models as well as blood temperature (°C) of different blood oxygen saturation levels (%). The different oxygenation levels of the blood sample are achieved via a perfusion oxygenator setup.^23^

Pig	Kidney (°C)	Lung (°C)	Heart (°C)	Brain (°C)	${\mathrm{sO}}_{2}$ (%)	Blood (°C)
1	31.3	32.5	35.0	35.7	82.0	37.1
2	33.8	33.3	35.7	36.1	92.8	36.9
3	35.1	32.8	34.5	33.1	98.6	37.3

A visualization of the captured data for the heart of the third animal is shown in Fig. 4. With the hyperspectral pushbroom camera, the largest field-of-view could be captured, followed by the multispectral 3×3-VIS camera and finally by the multispectral 4×4 VIS and 5×5-NIR cameras. With all four camera systems, the measuring object field given by the cursor can be successfully recorded.

**Fig. 5:**
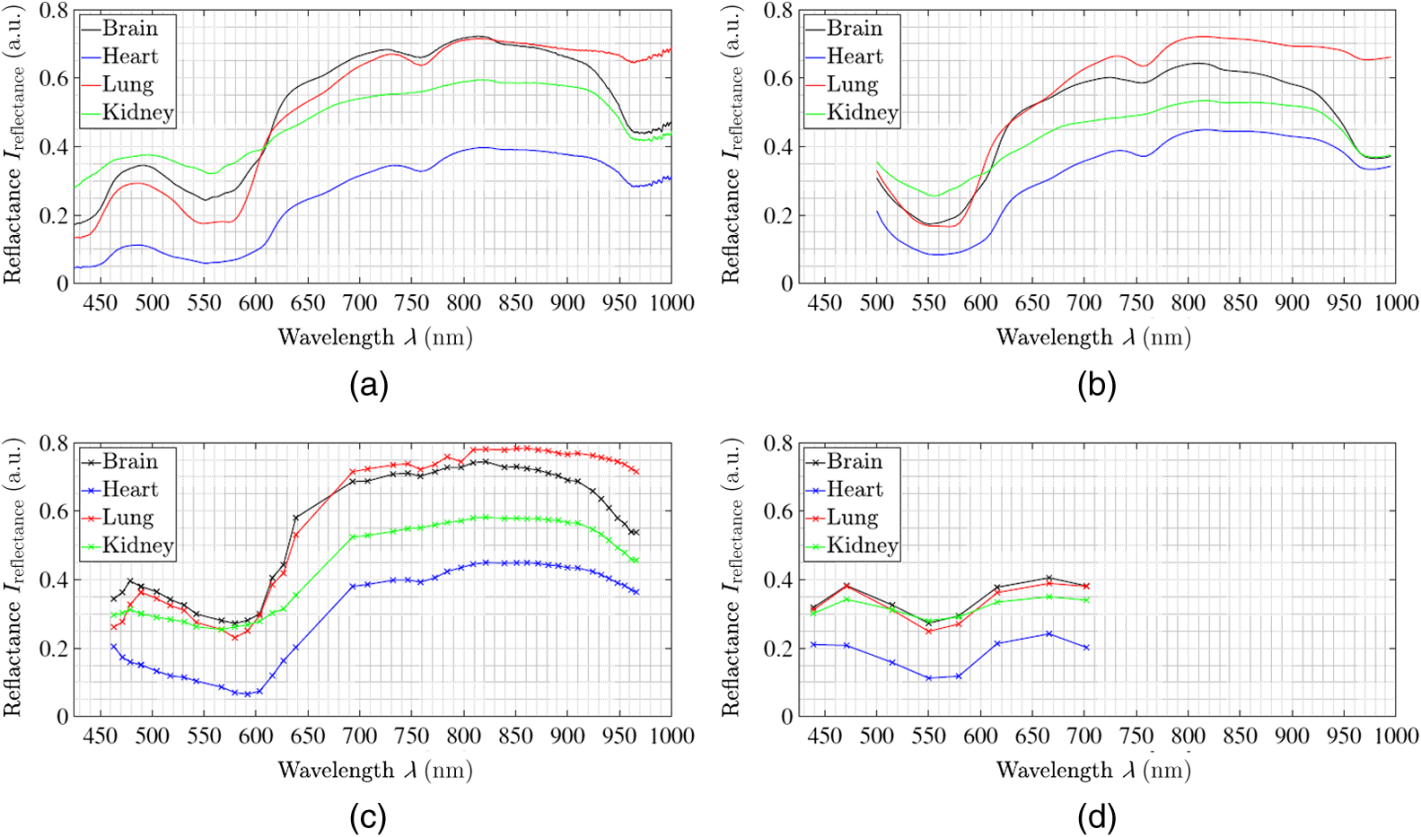
The spectral reflectance behaviors for all four analyzed organs averaged over all measurements presented of (a) spectrometer ground truth data, (b) hyperspectral pushbroom camera, (c) multispectral 41-band setup, and (d) multispectral 3×3 camera.

For each of the four porcine organs, a similar spectral behavior is captured through all four different acquisition methods in the corresponding spectral intervals of ∼450 up to 700 or 1000 nm, respectively (see Fig. 5). All organs show a local peak at about λ≈485  nm, a local minimum at about λ≈555  nm, and a rising edge in the range of λ≈600  nm to λ≈630  nm. The maximal reflectance intensity of the spectra is at λ≈800  nm. In addition, the different organs have specific spectral variations (see Fig. 5). The reflectance data averaged over all measurements results in an ∼50% lower reflectance intensity for the heart over the entire spectrum compared to the other three spectra in an overall constant setting for all organs. Further, the reflectance variation over the entire analyzed spectrum is smaller for the porcine kidney and porcine heart samples compared to the lung and brain samples. Brain and lung data are comparable with specific differences. The brain spectra shows an intensity decrease in the infrared (IR) range at λ=950  nm, whereas it has slightly higher intensity in the visible range (λ=460  nm to λ=600  nm) in comparison to lung data. The reflection spectra of the kidney are comparable to the brain and lung spectra and intersect them at λ=600  nm. It shows a flat trend in the NIR and IR range, with a decrease at λ=950  nm similar to the heart spectrum.

In detail, differences through the spectral behavior of the organs exist over the complete analyzed spectra. While heart, kidney, and brain show a different decrease of intensity starting at λ=950  nm, the spectrum of the lung remains constant. Thus the lung shows a strongly increased reflectance from about λ=950  nm compared to the other three organs. Furthermore, the rising band at λ≈600 to 630 nm is steep for heart, lung, and brain, whereas the increase in reflectance before and after that band is smooth. In addition, these organs show a differently pronounced local peak at λ≈730  nm and a local minimum at λ≈757  nm. Considering the kidney dataset, the spectrum results are smoother, with a monotonic increase in reflectance from λ≈560  nm up to λ≈685  nm. After this point, the reflectance remains relatively flat showing no pronounced local maxima or minima, different from the other organ samples.

All the obtained spectra are consistent with the spectral properties of common tissue chromophores in the range of 400 to 1000 nm. The primary light-absorbing compounds in tissue within this range are Hb and water. Depending on different oxygen conditions, a conformational change occurs in the Hb molecule, which leads to spectral changes in the UV–VIS–NIR range. The spectrometer data display in the range of λ=380 to 500 nm the extreme intense Soret bands.^36^ The minima at λ=555  nm and λ=765  nm in the reflectance curve are characteristic bands of the porphyrin Hb molecule.^36^ The flattening of the spectral curve from λ=600  nm to λ=740  nm as well as the formation of two minima in the range from λ=500  nm to λ=600  nm are spectral features of ${\mathrm{HbO}}_{2}$. For the lung data, the double peak of ${\mathrm{HbO}}_{2}$ is visible in the spectrometer, pushbroom, and 41-band data. The 3×3-VIS data contain too few λ-bands to resolve the characteristic spectral properties. The observed local minima at λ=760  nm and λ=970  nm correspond to water-specific spectral properties, e.g., the absorption peaks of water.^25^

**Fig. 6:**
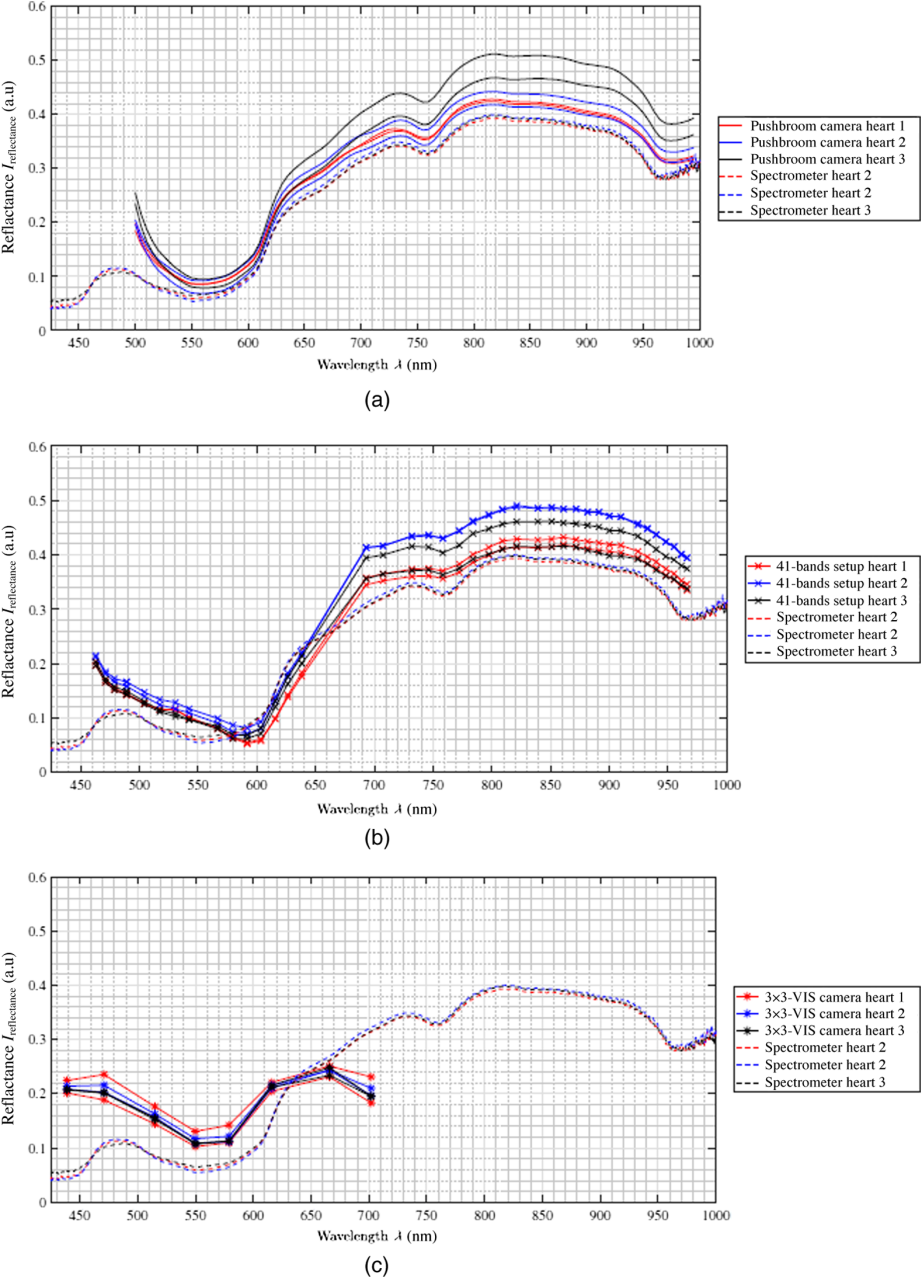
For the porcine heart measurements, all curves acquired by the different spectral cameras—(a) hyperspectral pushbroom camera, (b) 41-bands setup, and (c) 3×3-VIS camera—match the behavior of the reference spectrometer data (dashed lines). For each of the three camera acquisition methods, two ROIs are selected, reconstructed, and averaged over the three measurements. The resulting two ROIs are presented here.

**Fig. 7:**
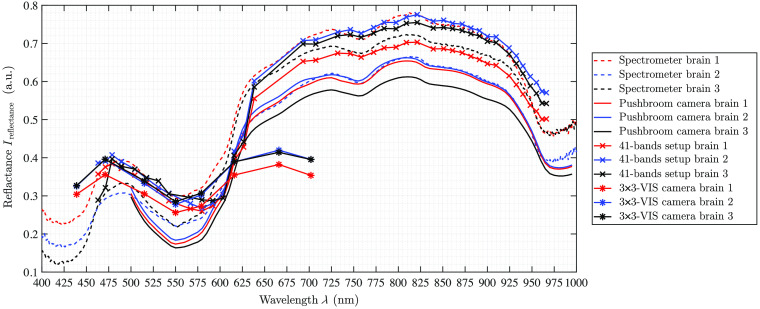
Porcine brain measurements of all three pigs both two selected ROIs are averaged to one spectral curve.

**Fig. 8:**
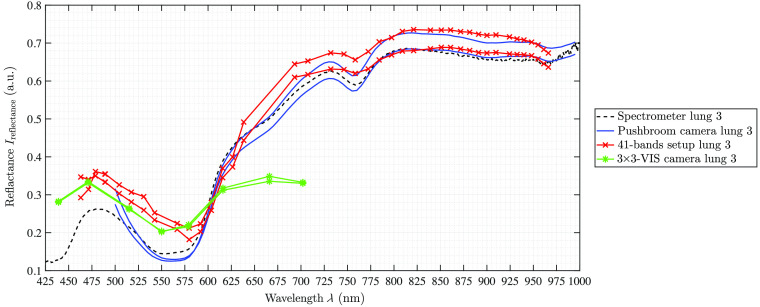
Porcine lung measurements of the third pig at two different selected ROIs. These spectral curves are representative for the other two pigs as well.

**Fig. 9:**
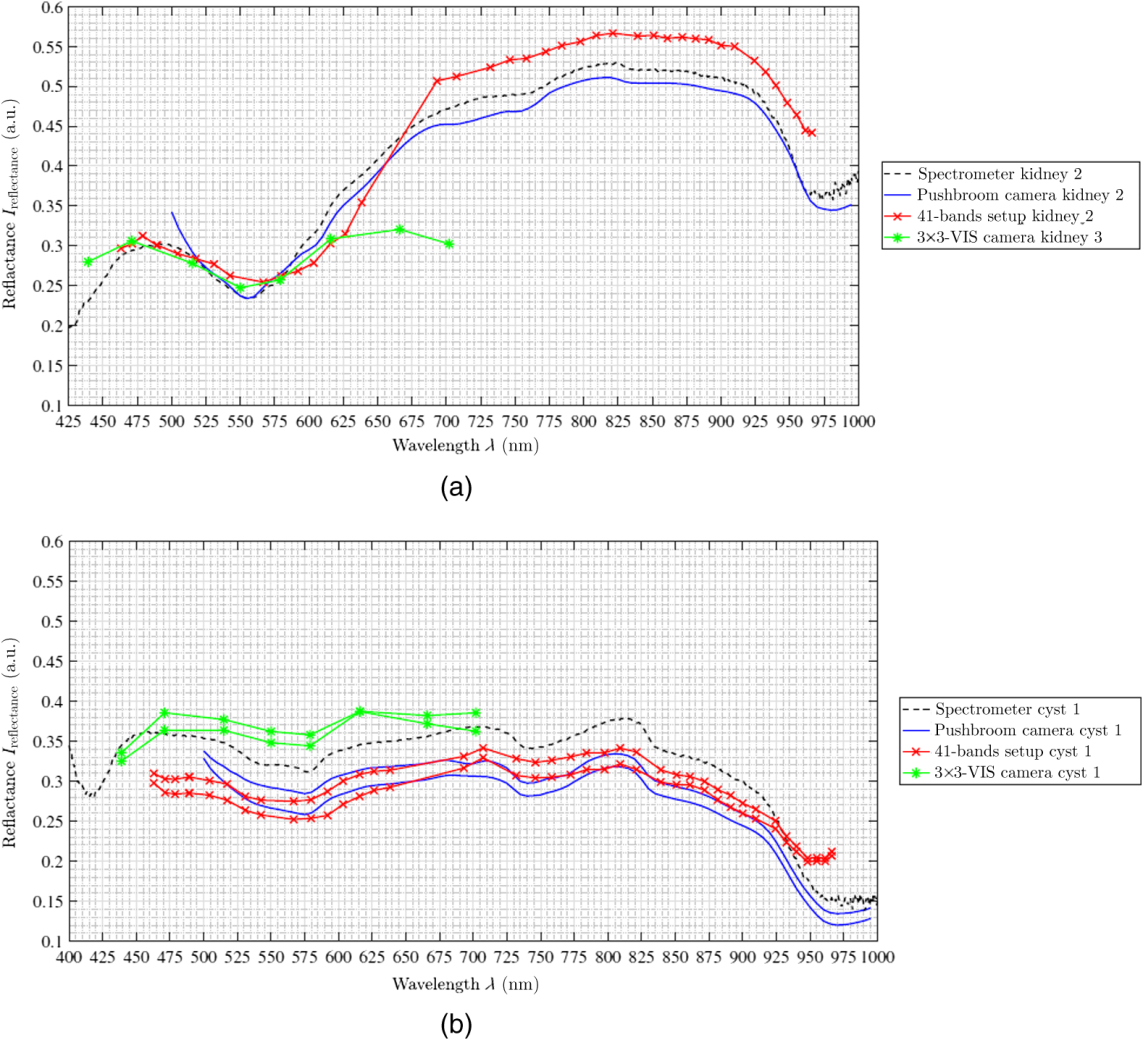
Porcine kidney measurements of (a) healthy kidney tissue from the second pig and (b) kidney with a superficial cyst from the first pig, both averaged over both measured ROIs. The spectral curves in (a) are representative for the other two pigs as well, which are omitted for readability. In (b), the spectrometer data of the healthy kidney of the first pig are plotted for comparability.

Both the individual organ spectra of the camera measurements and the spectrometer measurements vary in relation to the reflectance intensity (i.e., intensity axis in Figs. 5–9). This behavior can also be detected in the spectrometer reference measurement of the three pigs. However, the measured and reconstructed 41-bands setup reflectance [cf., Fig. 5(c)] and the reflectance of the pushbroom camera [cf., Fig. 5(b)] lie within these reference ranges and correspond to the optical behavior of the organ. To quantify the similarity between the spectra of the spectral cameras and the spectra of the reference a normalized cross correlation (NCC), i.e., Pearson correlation coefficient, is applied (see Table 3). All NCC metrics are in the range of 0.97 up to 0.99 for the pushbroom camera and 41-bands setup data. The difference of about 1.5% for NCC values between the comparisons of spectrometer and pushbroom or 41-bands setup (0.995 versus 0.980) comes along the linear fitting between the single bands with wider band distances for the 41-bands setup data. In addition, the comparisons between the single camera setups are performed to characterize the comparability between the three different acquisition setups. The Pearson coefficients between pushbroom and 41-bands setup are in the range of 0.985±0.005, which shows a robust comparability between these two setups.

**Table 3:** NCC between the averaged spectral curves of the different acquisition methods. The curves of the pushbroom, the 41-bands, and the 3×3-VIS camera setup are linear fitted between the single bands. The number of data points indicates the used points for NCC calculation.

Camera comparison	Kidney	Lung	Heart	Brain	λ-area (nm)	Points
Spectrometer versus pushbroom	0.9938	0.9978	0.9957	0.9982	500 to 995	1518
Spectrometer versus 41-bands	0.9707	0.9822	0.9817	0.9820	460 to 970	1540
Spectrometer versus 3×3-VIS	0.8369	0.8397	0.5894	0.8533	400 to 700	850
Pushbroom versus 41-bands	0.9782	0.9841	0.9870	0.9824	500 to 970	1434
Pushbroom versus 3×3-VIS	0.9408	0.9718	0.8975	0.9566	500 to 700	579
41-bands versus 3×3-VIS	0.7378	0.7621	0.7376	0.8250	460 to 700	685

The measured and reconstructed reflectances of the 3×3-VIS camera [cf., Fig. 5(d)] are different to the other two camera acquisition methods. The captured spectral data are located exclusively in the VIS wavelength range between λ=425  nm to 700 nm. The first six wavelength bands in the range of λ=425 to 625 nm correspond to both the spectrometer reference data and the other two camera systems. Only for the heart measurements (cf., Fig. 6), the reflectance intensity of the first band is too high and do not fit the reference. Furthermore, the last two measured and reconstructed wavelength bands at approx. λ=665  nm and λ=700  nm show consistently too low reflectance intensities for all organs (cf., Figs. 5–9).

The NCC results of the 3×3-VIS camera data substantiate these appearances (see Table 3). When comparing 3×3-VIS camera with the reference spectrometer, the Pearson coefficient reaches 0.84±0.01 for kidney, lung, and brain measurements and 0.59 for heart measurements. Due to the data fitting for the pushbroom setup as well as for the 3×3-VIS camera setup, the NCC is higher when comparing these setups (0.90 up to 0.97). Compared to the multispectral 41-bands setup, the NCC data are reduced, in a range of 0.74 to 0.83, as for both acquisition methods less data points are used for fitting.

The spectra of the porcine hearts are similar for all different recordings methods, i.e., spectrometer and different setups, and through all different measurements, shown in Table 3 and Fig. 6. The first data points of the spectra of the hyperspectral pushbroom camera (λ≈500 to 515 nm) as well as the first two spectral bands of the 4×4-VIS camera of the 41-bands setup show a strong scattering or inaccurate behavior. This is visible for all measurements across all organs (cf., Figs. 7 and 8) for brain of all three pigs and lung of the third pig, respectively. Further, the 41-bands dataset consists of a large gap in the spectral range of λ=640  nm and λ=690  nm. Therefore, in this region, the spectral behavior cannot be represented correctly, as this gap is the transition between the two combined multispectral cameras.

Especially, the multispectral snapshot cameras show some discontinuities in the reconstructed reflectance bands, as already described for the first two spectral bands of the 4×4-VIS of the 41-bands setup. Further, the band at λ=630  nm always underestimates the reflectance intensity. In contrast, all bands of the 5×5-NIR represent the spectral organs behaviors with all local maxima and minima precisely.

In addition to the healthy porcine organs, one kidney affected with a cyst has been evaluated (see Fig. 9). This affected kidney is from the first pig and has been analyzed 3.75 h after organ collection with an organ core temperature of $T_{\text{core}}=37.0{}^{\circ}C$. Compared to the healthy kidney tissue of the first pig [cf., Fig. 9(a)], the spectral behavior of the cyst is completely different [cf., Fig. 9(b)], as a cyst contains a hydrous solution with cell components as well as hormones and electrolytes but almost no blood. This highly affects the spectral behavior. Between λ=425 to 900 nm, the spectrometer cyst data are in the range of $I_{\text{reflectance}}=0.35$. The local minimum at λ=580  nm is visible in all camera setup data. Further, the local maxima at λ=710  nm and λ=815  nm as well as the local minimum at λ=740  nm are formed by the pushbroom and the 41-bands setup. The NCC between the healthy kidney data and cyst data is 0.0385, which shows no relevant spectral correlation between these two types. Compared to the ground truth spectral cyst data of the spectrometer, the intensities of spectral data of the pushbroom camera as well as the 41-bands setup are slightly reduced. According to the NCC, the spectral curves of the pushbroom camera (0.996) and the 41-bands setup (0.930) are in good correlation with the reference spectra. Further, both setups are almost overlapping and comparable to each other with NCC of 0.926. The spectral intensity data of the 3×3-VIS are slightly increased compared to the spectrometer data but follow the spectral trend as well.

### 3.3 Blood Measurements

The blood has been included into the blood circuit after t=6.75  h of storing on ice. The first blood measurement has started t=7  h after storing. The temperatures of the blood sample during the measurements are presented in Table 2. The averaged blood temperature during all measurements has been $T_{\text{core}}=37.1{}^{\circ}C$.

**Fig. 10:**
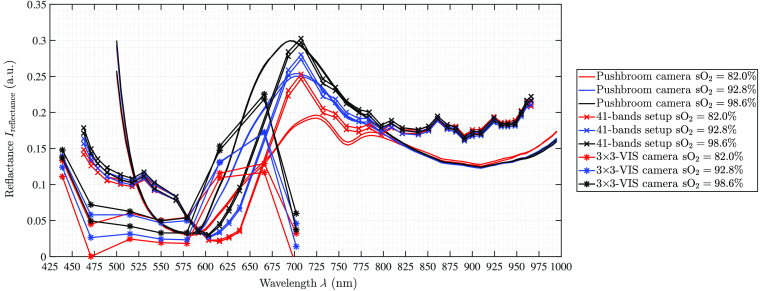
Blood measurements of all three camera setups. For each setup, the two ROIs average over the three measurements are presented.

The blood spectra show the expected behavior. There is a dependence on the degree of oxygenation of the blood with the reflectance characteristics. As shown in Fig. 10, the oxygenated blood curve has higher intensities in the near-IR ranging from λ=600 to 800 nm for the acquisition options of the hyperspectral pushbroom camera and the multispectral 41-bands setup. The multispectral 5×5-NIR data of the 41-bands setup show higher variability along the spectral curve compared to the hyperspectral pushbroom blood data as well as compared to the spectral trends in the 5×5-NIR organ measurements.

The individual behaviors for the different oxygenation levels with the characterized local minimum at λ=760  nm for blood with low oxygen saturation is clearly visible for the pushbroom camera data. The 41-bands data follow the same trend, but it is less pronounced due to only 41 bands. The 3×3-VIS camera data only cover the visual range and follow the expected blood spectra trend for the eight bands until λ=675  nm. The λ=700  nm band is far too low in terms of reflectance intensity. For the sixth (λ=615  nm) and seventh band (λ=665  nm) of this camera, the differentiation between oxygenated and deoxygenated blood is visible. This has been also expected for the eighth band (λ=700  nm) but could not be proven from the data. No spectrometer data have been analyzed for the blood measurements as the spectrometer data have shown total reflections, which omit the spectral behavior of the different blood samples.

## 4 Discussion

This study compares different spectral cameras for a possible clinical use, such as organs surveillance during transplantation by monitoring organ and blood specific spectral features. The performance of the camera systems has been evaluated using a reference spectrometer, which is validated using a standardized ColorChecker chart. The measured spectrometer data fit to the validation data with deviations up to about 5% due to the fact that the validation data are measured data by other authors.^34^^,^^35^ With respect to published as well as own measurement deviations, the data fit well. Further, the calculated Pearson coefficient represents a very high correlation. Thus the spectrometer data are used as reference system in this study.

In comparison with the reference system, two of the three camera setups show good results with regard to the reproduction of spectral information. These results could be obtained for measurement objects of different application areas: (1) standardized non-biological material and (2) complex biological materials. Both, in the analysis of the ColorChecker as well as the organs, the hyperspectral pushbroom camera (0.999 and 0.996, respectively) and the multispectral 41-bands setup (0.979 as well as 0.979) show a very high positive correlation with the ground truth data of the spectrometer. The results for complex biological material, i.e., different organs, in the visible and NIR wavelength range, i.e., λ=515 to 995 nm for the pushbroom camera and λ=470 to 960 nm for the 41-band setup, are valid compared with published tissue spectra.^37^ The measured and calculated reflectance in the stated λ-ranges reproduces the real optical behavior and can show relevant tissue characteristics as Hb- and water-specific properties. This applies for the pushbroom camera as well as for both cameras of the 41-band setup. The better reproducibility of the specific reflectance for the pushbroom setup is caused by the higher spectral resolution.

In terms of reconstruction of the reflectance, the 3×3-VIS camera is comparable to the 4×4-VIS camera of the 41-band setup but with less λ-bands. It becomes apparent that eight wavelength bands are not sufficient for a synchronized and comprehensive supervision of all specific organ markers such as HbO or water bands. This is manifested by the inaccurate representation of relevant peaks of the tissue chromophores, e.g., it will not become possible to detect relevant peak differences between Hb and ${\mathrm{HbO}}_{2}$ or other possible shifts in characteristic Q bands of present porphyrin complexes. For lung imaging, a double peak at about λ=555  nm in the data shows the presence of ${\mathrm{HbO}}_{2}$ (see Fig. 8). Only in the 3×3-camera data, this double peak is not visible due to the few spectral bands. The multispectral 4×4-VIS camera holds 16 bands in a smaller wavelength interval as the multispectral 3×3-VIS camera, which at least are needed to detect small specific changes in the spectral appearance.

However, the 3×3-camera data behave similar to the 4×4-camera and pushbroom camera data for the first six wavelength bands, which are too few bands for wide spectral analysis. The last two bands at λ=665  nm and λ=700  nm always underestimate the reflectance intensity. It is not clear for what reason these two bands produce such false values. One reason could be a faulty correction matrix, which need further inspection. Another reason for that behavior could be due the lower transmission spectrum for that bands, which results in a lower signal-to-noise ratio (SNR) of these bands compared to the other bands. Such a low SNR also appears for the first data points of the hyperspectral pushbroom camera (λ≈500 to 515 nm) and the first two spectral bands of the multispectral 4×4-VIS camera. However, the low SNR of the first data points of the hyperspectral pushbroom as well as the multispectral 4×4-VIS camera are caused by the low illumination intensity in that spectral interval due to the quartz-tungsten-halogen spectrum.^10^^,^^14^^,^^29^

Despite the fact that the 41-bands setup has a lower spectral resolution than the pushbroom camera, both camera setups are suitable for identifying different physiological tissue properties like blood or other markers in the analyzed tissue sample or unphysiological organ structures like cysts on the kidney [cf., Fig. 9]. The higher spectral resolution of the pushbroom camera is at the cost of a longer recording time $t_{\text{record}}=6\;s$, whereas the multispectral cameras allow real-time imaging. Although this study has been recorded with 20 fps, it is readily possible to use the study’s setup with higher frame rate (e.g., 25 or 60 fps) and lower exposure settings. The reduced spatial resolution of multispectral snapshot cameras can be compensated using a suitable demosaicing algorithm.^38^

For the 41-bands setup data and the pushbroom camera data, it is visible that only the healthy kidneys have been purged of blood. The organs brain, heart, and lung show the local minimum at about λ=760  nm, which indicates the presence of blood. The kidney cyst data show the presence of blood as well. The varying expression of this local minimum indicates a different blood content in the analyzed organs. All setups can show the spectral variability of various organ types, which are caused by the different chemical and morphological compositions. For example, the expected reduction of reflectance by 50% for the heart compared to the other organs is clearly presented by all setups, as muscle tissue holds more chromophore than other tissue resulting in stronger absorbance.^39^.

The variation behavior of different reflectance intensity levels for different samples of the same type of organ or different ROIs within one organ sample is expected, as the reflectance intensity is also affected by the WD as well as illumination distance. Normally, this effect is compensated using the correction pipeline as proposed by Wisotzky et al.^10^ and Eq. (1). This includes the white reference image $I_{\text{white}}$, which is acquired using a flat calibration board, whereas the organs show a topological 3D structure causing deviations of the illumination and WD over the whole organ structure. Further, different organs of the same type are always of a slightly different anatomical texture and thus show small variations in spectral behavior. Therefore, the reconstructed reflectance intensity is dependent on the different organ samples as well as on the selected ROI (see Figs. 6 and 7). This difference is clearly visible in Fig. 7 as a larger gap between the 41-bands setup data and the pushbroom camera data, due to different specular reflections in the images and therefore different selected ROIs.

Another reason for differences in reflectance intensity can be variant tissue temperature. The occurring temperature differences arising in this study are caused by different types of tissue, different densities, unequal organ weights, and sizes but do not significantly effect the results. We have started analyzing the organs of animal three and finished with animal one. This means, the time the organs are stored on ice has been in the range of 1 h for the brain of animal three up to 3.5 h for the heart of animal one. This temporal difference could affect the reflectance properties of each organ and tissue,^40^^–^^42^ which could explain the small discrepancies between the same organ types of different pigs. However, this aspect has not been the subject of this study and has not been analyzed in detail.

In general, both acquisition methods, pushbroom and snapshot setups, allow an exact reproduction of the physiological markers and pathological conditions of the analyzed anatomical structures. The precision of these representations is dependent of each setup, meaning the choice of the optimal setup would be dependent on the specific clinical aim. Different tissue parameters such as oxygen saturation, water, or blood concentration could be derived using all setups, whereas a higher number of spectral bands is preferable for a snapshot setup. Thus both basic acquisition principles could be feasible for clinical tissue analysis and differentiation or organ surveillance, e.g., to differentiate between single tissue structures as kidney, lung, or muscle, as well as to differentiate between healthy and pathological areas within one tissue type or organ.

To further verify the validity and reliability of the results obtained in this study, experiments on other biological objects or other organs of origin, i.e., other animal organs such as those from cattle or actual human tissue, would be useful.

## 5 Conclusions

In this work, we have presented and analyzed the possible use of three different spectral acquisition methods for intraoperative organ surveillance. All camera setups are able to reconstruct the spectral behavior of the analyzed organs in its available wavelength range. For accurate and robust analysis of clinically relevant biological materials, fine scanning of the analyzed spectral range is essential. Therefore, the hyperspectral pushbroom camera is the most accurate having spectral resolution of 5 nm. However, with a minimum of 6 s recording time, it does not allow real-time monitoring. The setup with two multispectral 4×4-VIS and 5×5-NIR cameras represents a good compromise. It scans the wavelength range with 41 bands efficiently with a spectral resolution of ∼12.5  nm and allows real-time imaging as well as accurate analysis of the VIS–NIR range. Thus several tissue chromophores and indices can be analyzed in the range of 400 to 1000 nm to classify the tissue and its quality. Disadvantages of this camera setup is the small spectral gap at λ=675±20  nm and the requirement of two cameras. The multispectral 3×3-VIS allows a better spatial resolution of the data but has the lowest number of bands. As a result, spectra cannot be reconstructed properly and physiological or pathological changes cannot be detected robustly.

Altogether, knowledge of the suitability of spectral camera systems to determine the exact chemical properties of a biological object could offer a promising opportunity to measure organ- or blood-specific parameters. In combination with previously published literature about optical organ and tissue behaviors,^37^^,^^39^^,^^43^^–^^47^ this study allows the selection of the optimal HMSI system for the use for a specific medical applications.^12^^,^^31^^,^^48^^,^^49^
