# Supplementary material for: Comparison of different spectral cameras for image-guided organ transplantation
Source: J Biomed Opt. 2021 Jul 24;26(7):076007. doi: 10.1117/1.JBO.26.7.076007 (PMC8305772; doi:10.1117/1.JBO.26.7.076007)
Supplement: Supplementary file 1 [file JBO_026_076007_SD001.pdf]

1 Supplementary Material

Table S1: This table shows the spectral peak and FWHM of each band for all used snapshot cameras and filters. The bands are sorted, starting with lowerst λ, and the numeration does not correspond to the index positions on the sensor.

	Band #	0	1	2	3	4	5	6	7	8	9	10	11	12
3x3	λ [nm]	424	457	497	534	561	599	640	680					
Camera	FWHM	51	45	36	32	30	29	27	26					
4x4	λ [nm]	463	471	478	489	490	504	518	531	543	567	580	592	603
Camera	FWHM	10.8	15.5	20.2	10.8	12.1	10.8	9.4	7.4	7.4	7.4	7.4	7.4	10.8
5x5	λ [nm]	693	707	732	746	758	772	784	798	809	821	839	851	861
Camera	FWHM	4.0	4.4	5.1	4.7	7.1	6.8	6.8	8.5	6.8	7.1	10.1	8.1	8.5

	Band #	13	14	15	16	17	18	19	20	21	22	23	24
4x4	λ [nm]	616	626	638									
Camera	FWHM	13.4	15.5	15.5									
5x5	λ [nm]	872	881	891	900	910	924	932	940	948	955	961	966
Camera	FWHM	8.8	10.9	12.2	15.0	12.9	15.3	14.2	18.7	19.4	25.0	17.7	15.3
